# High‐Voltage Instability of Vinylene Carbonate (VC): Impact of Formed Poly‐VC on Interphases and Toxicity

**DOI:** 10.1002/advs.202305282

**Published:** 2023-11-08

**Authors:** Maximilian Kubot, Lisa Balke, Johannes Scholz, Simon Wiemers‐Meyer, Uwe Karst, Heiko Hayen, Hyuck Hur, Martin Winter, Johannes Kasnatscheew, Sascha Nowak

**Affiliations:** ^1^ MEET Battery Research Center University of Münster Corrensstraße 46 48149 Münster Germany; ^2^ Institute of Inorganic and Analytical Chemistry University of Münster Corrensstraße 48 48149 Münster Germany; ^3^ R&D Campus Daejeon LG Energy Solution 188, Munji‐ro, Yuseong‐gu Daejeon 34122 Republic of Korea; ^4^ Helmholtz‐Institute Münster IEK‐12 Forschungszentrum Jülich GmbH Corrensstraße 46 48149 Münster Germany

**Keywords:** electrode crosstalk, electrolyte additive, electrolyte oxidation, high‐voltage, HPLC‐MS, LDI‐ToF‐MS, Li ion battery, poly‐vinylene carbonate, structure elucidation, toxicity, vinylene carbonate

## Abstract

Full exhaustion in specific energy/energy density of state‐of‐the‐art LiNi_x_Co_y_Mn_z_O_2_ (NCM)‐based Li‐ion batteries (LIB) is currently limited for reasons of NCM stability by upper cut‐off voltages (UCV) below 4.3 V. At higher UCV, structural decomposition triggers electrode crosstalk in the course of enhanced transition metal dissolution and leads to severe specific capacity/energy fade; in the worst case to a sudden death phenomenon (roll‐over failure). The additive lithium difluorophosphate (LiDFP) is known to suppress this by scavenging dissolved metals, but at the cost of enhanced toxicity due to the formation of organofluorophosphates (OFPs). Addition of film‐forming electrolyte additives like vinylene carbonate (VC) can intrinsically decrease OFP formation in thermally aged LiDFP‐containing electrolytes, though the benefit of this dual‐additive approach can be questioned at higher UCVs. In this work, VC is shown to decrease the formation of potentially toxic OFPs within the electrolyte during cycling at conventional UCVs but triggers OFP formation at higher UCVs. The electrolyte contains soluble VC‐polymerization products. These products are formed at the cathode during VC oxidation (and are found within the cathode electrolyte interphase (CEI), suggesting an OFP electrode crosstalk of VC decomposition species, as the OFP‐precursor molecules are shown to be formed at the anode.

## Graphical abstract

Illustration of the experiment to investigate possible organofluorophosphates–(OFP) interphase incorporation due to oxidative degradation of vinylene carbonate (VC)‐containing electrolytes by means of different mass spectrometric methods. The findings illustrate in operando formation of poly‐VC at high‐voltages for the first time and also support the thesis of OFP incorporation into the solid electrode interphase during battery cell operation.

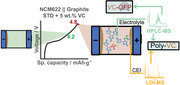

## 1 Introduction

The recent legislation of the European Parliament toward zero CO_2_ emissions for mobility and commercial vehicles from 2035 triggers the urgency for alternative fuels and energy sources.^[^ ^1^ ^]^ The development of state‐of‐the‐art (SOTA) lithium‐ion batteries (LIBs) experiences even greater attention as the demand for portable, stationary, and automotive energy storage solutions with high specific energy/energy density rises further. Still, the specific energy of LIBs is limited and in practice not fully exhausted for stability, safety, and cycle life reasons.^[^ ^2^, ^3^, ^4^ ^]^

The LIB is commonly composed of a layered transition metal oxide (e.g., LiNi_x_Co_y_Mn_z_O_2_ (NCM; *x* + *y* + *z* = 1))‐based cathode and a carbonaceous anode.^[^ ^5^, ^6^ ^]^ The SOTA electrolyte is the conducting salt LiPF_6_ in a mixture of a cyclic carbonate, that is ethylene carbonate (EC), and a linear carbonate, for example, ethyl methyl carbonate (EMC), frequently optimized with functional electrolyte additives like vinylene carbonate (VC).^[^ ^7^, ^8^, ^9^ ^]^ These types of electrolytes possess a high enough ionic conductivity over a wide temperature range and can effectively passivate the Al current collectors against anodic dissolution.^[^ ^10^, ^11^, ^12^ ^]^ Also, they effectively passivate the highly reductive anode by forming the so‐called solid electrolyte interphase (SEI), thus enabling electrode operation at ≈0 V versus Li|Li^+^,^[^ ^13^, ^14^, ^15^, ^16^, ^17^, ^18^, ^19^ ^]^ and are thermodynamically and/or kinetically stable at conventional electrode potentials of NCM‐cathodes, which are typically operated <4.3 V versus Li|Li^+^.^[^ ^3^, ^20^, ^21^, ^22^, ^23^, ^24^, ^25^, ^26^ ^]^

However, there are still challenges posing substantial concerns regarding the safety and durability of these electrolyte systems. Besides the safety concerns related to the flammable carbonate‐based solvents, including high vapor pressure and low flashpoint (linear carbonates),^[^ ^8^, ^27^, ^28^, ^29^ ^]^ LiPF_6_ raises major concerns in terms of toxicity via degradation products, that is organofluorophosphates (OFPs). LiPF_6_, being highly sensitive toward hydrolysis, can react with trace amounts of water to form POF_3_ and hydrofluoric acid (HF), which further react with the organic solvents to finally form OFPs and organophosphates (OPs).^[^ ^30^, ^31^, ^32^, ^33^, ^34^ ^]^ The toxicity of OFPs stems from the P─F bond irreversibly interacting with the catalytically active site of the enzyme acetylcholine esterase (AChE).^[^ ^35^, ^36^ ^]^ The inhibition of the AChE results in a permanent stimulation of the neuromuscular junctions leading to paralysis or death. However, Henschel et al. illustrated a negligible toxicity of acidic OFPs, that is, those with a free hydroxyl group located at the phosphorus, consequently suggesting a relation between sidechains of OFPs and toxicity.^[^ ^37^ ^]^

The specific energy/energy density and theoretical specific capacity in LIBs are currently limited for structural stability reasons. With SOTA NCM cathodes, capacity utilization is <70%, realized via adjustment of the upper cut‐off voltage (UCV) to a maximum of ≈4.3 V; depending on the detailed NCM composition.^[^ ^25^, ^38^, ^39^, ^40^ ^]^ Though higher UCVs would increase specific energy by increasing both, the mean discharge voltage and specific capacity, associated increase in cathode instability will decrease cycle life due to structural instability‐caused inactivity, resistance rise, and accompanied transition metal (TM: Ni, Co, Mn) dissolution. Through electrode crosstalk, TMs would then deposit at and thus damage the anode.^[^ ^41^, ^42^, ^43^ ^]^ TMs locally enhance resistance on the anode and trigger inhomogeneous Li deposition/plating, which results in capacity losses and enhanced risk of short circuits via the formation of high surface area lithium (HSAL), for example, Li dendrites.^[^ ^44^, ^45^, ^46^ ^]^ The additive lithium difluorophosphate (LiDFP) was found to prevent the deposition of TMs on the anode by scavenging them before they can reach and damage the anode, resulting in a longer cycle life even at UCVs >4.3 V.^[^ ^47^ ^]^ However, LiDFP is also a direct precursor of OFPs and leads to safety concerns for commercial applications. In our recent study, we identified an elevated amount of OFPs and a new class of oligo‐FPS in thermally aged LiDFP‐containing electrolytes.^[^ ^48^ ^]^ However, it was shown that with the addition of film‐forming electrolyte additives (e.g., VC), the OFP formation and thus the toxicity of the electrolyte can be decreased. Therefore, a comprehensive investigation of this phenomenon, especially within the LIB and during battery operation is needed. However, the simplicity of in vitro analysis of thermally aged electrolytes is lost when analyzing the electrochemical degradation and the aforementioned suppression of OFP formation. Here, several new challenges arise when working with electrolytes extracted from an LIB as, for example, the anode and cathode are involved in any reactions taking place within the battery cell, as well as the reactivity of the electrolyte being highly dependent on the UCV. Further, as the SEI film formation is still a not fully understood phenomenon, where different processes are taking place simultaneously, the complexity of its composition rapidly reaches a point where surface analysis techniques (e.g., laser desorption/ionization‐time of flight mass spectrometry (LDI‐ToF‐MS)) reach their limit due to increasing ion suppression effects.

Electropolymerization reactions have been discussed for several classes of electrolyte additives, for example, isocyanates^[^ ^49^, ^50^, ^51^ ^]^ or vinyl compounds.^[^ ^52^, ^53^, ^54^, ^55^ ^]^ Postulated by Gasteiger et al., the oxidative degradation of VC results in poly‐VC, similar to the reductive degradation products that form the SEI.^[^ ^56^, ^57^, ^58^ ^]^ With the number of possible reactions increasing at high voltages, early termination of the polymerization with other reactive species should be possible, resulting in smaller and soluble VC_n_ molecules. These can be analyzed by means of high‐performance liquid chromatography‐mass spectrometry (HPLC‐MS) to elucidate the possible degradation products. As this oxidative degradation of VC takes place at the cathode side, the expected degradation products would most likely be observed in the cathode electrolyte interphase (CEI).^[^ ^59^ ^]^ While the SEI rapidly reaches a degree of complexity, which is difficult to analyze, the complexity of the CEI is lower. As the amount and relevance of VC oxidation is significantly higher and, compared to the STD electrolyte, can be already detected electrochemically during the first charge, VC should be the main reactant being oxidized at a UCV of 4.8 V (EC:EMC‐based electrolytes show no electrochemical oxidation plateau below ≈5.5 V *vs* Li/Li^+^), most of the products observed within the CEI should be VC‐derived.^[^ ^20^ ^]^ Thus, surface analysis of the CEI should be possible.

In this work, the oxidation onset of VC at UCVs >4.7 V was purposely triggered to initialize the proposed polymerization, that is, the formation of VC_n_‐molecules,^[^ ^20^ ^]^ with the aim to a) investigate the postulated poly‐VC generation through VC oxidation by means of HPLC‐MS, therefore to gather a deeper understanding of the film‐forming process, to b) obtain insides into the chemical compositions of the SEI/CEI and investigate the FP‐incorporation within the polymers of the SEI/CEI by means of LDI‐ToF‐MS.

## 2 Results and Discussion

**Figure 1:**
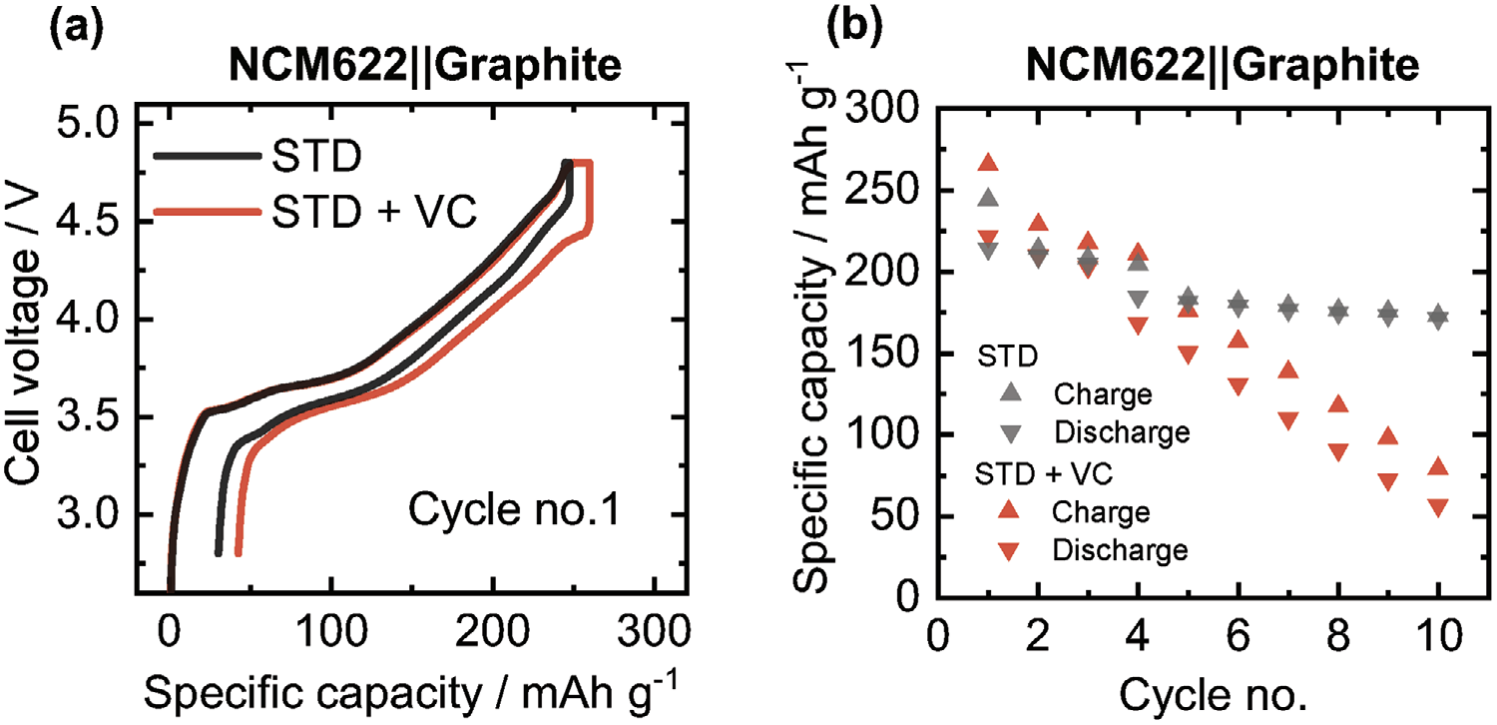
a) Voltage profile of first charge/discharge cycle with STD and VC‐containing electrolyte (5 wt.%) in NCM622||Graphite cells in the voltage range of 4.8–2.8 V (17.8 mA g^−1^; ≈0.1 C). b) The specific capacity during the initial ten charge/discharge cycles.

The first charge/discharge cycle for the LiNi_0.6_Co_0.2_Mn_0.2_O_2_ (NCM622)||graphite cell and state‐of‐the‐art/standard (STD) electrolyte is depicted in **Figure** **1**a for the enhanced UCV of 4.8 V in comparison with a VC‐containing electrolyte (5 wt.%). Higher specific charge capacity as well as specific capacity loss, suggest enhanced oxidative decomposition of VC, being in line with literature findings.^[^ ^20^ ^]^ The following charge/discharge cycles for both electrolytes are depicted in Figure 1b. The higher specific capacity losses (difference between charge and discharge capacity), and enhanced specific capacity fade additionally demonstrate the incompatibility of VC‐containing electrolytes for high‐voltage applications (UCVs > 4.7 V) from the battery‐performance point of view, for example for Mn/Li‐rich cathodes.^[^ ^60^ ^]^

**Figure 2:**
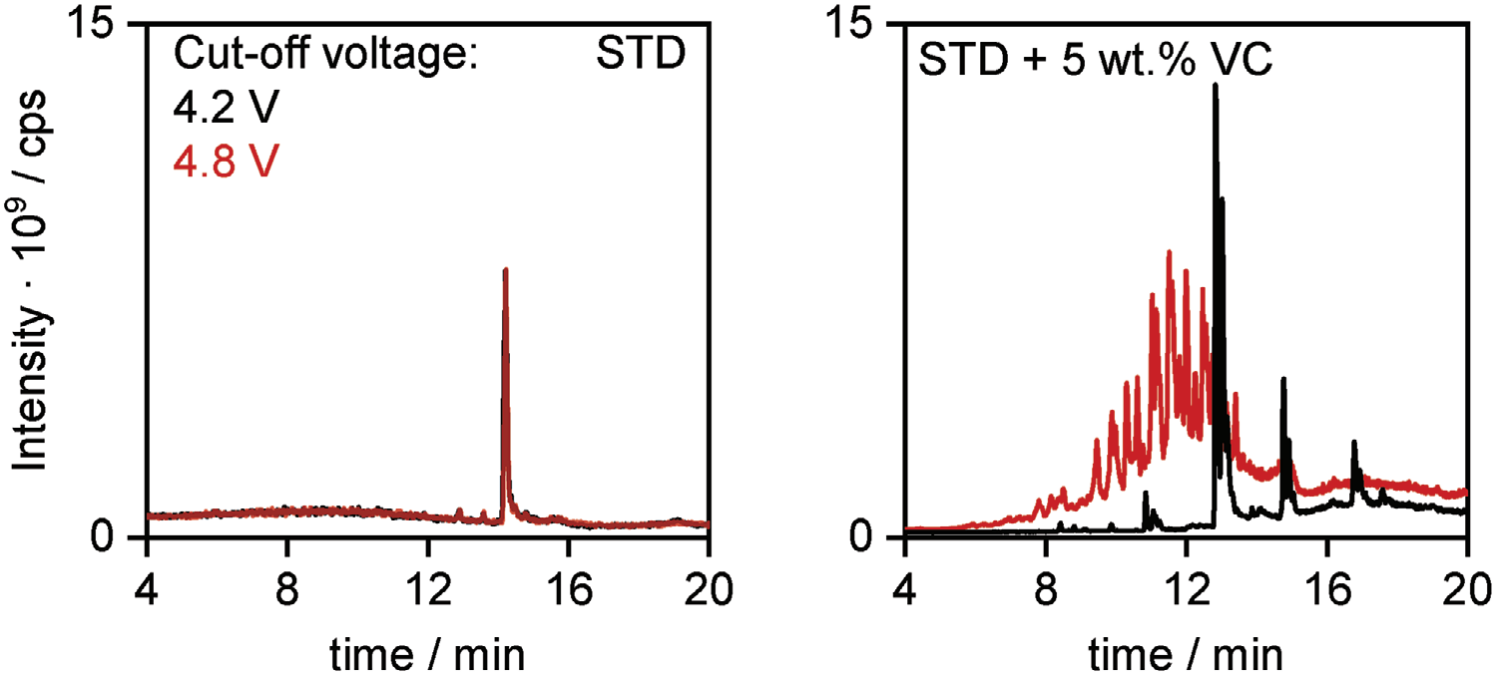
Total ion currents (TICs) obtained via HPLC‐MS analysis of the electrochemically aged STD electrolyte and STD + 5 wt.% VC operated at 4.2 V (black) and 4.8 V (red) UCV for 10 charge/discharge cycles.

HPLC‐MS analysis reveals an elevated number of compounds generated at UCVs >4.7 V, in the course of VC oxidation (**Figure** **2**). No difference is observed at low and high UCVs (4.2 and 4.8 V) for the STD electrolyte, suggesting its stability, while the VC‐containing electrolyte shows a variety of decomposition products at 4.8 V.

### 2.1 Polyvinylene Carbonates as Oxidative Reaction Products

**Figure 3:**
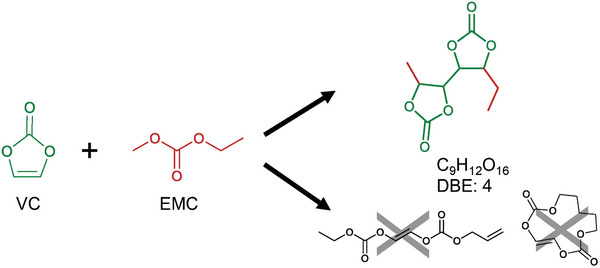
Evaluation of possible oxidative reaction products of VC and EMC regarding their DBE. The proposed structure is observed as a direct degradation product in HPLC‐MS measurements. The crossed‐out structures represent theoretically possible structures that, however, are not likely to form within the LIB electrolyte during battery operation.

One class of identified compounds is of high interest due to their, for typical electrolyte degradation products, unusually high number of double bond equivalents (DBE) that are not explicable with literature‐observed classes of degradation products elucidated by mass spectrometry. The known products include among others: polycarbonates, polyglycols, monocyclic ring structures, and organo(fluoro)phosphates.^[^ ^37^, ^61^, ^62^, ^63^, ^64^, ^65^, ^66^, ^67^, ^68^, ^69^, ^70^ ^]^ None of the above gave a satisfying solution to the found molecules. Here, DBEs are observed which strongly indicate the presence of polycyclic ring structures when using VC as a film‐forming additive. As postulated by Gasteiger et al., these polycyclic compounds should derive from VC itself (poly‐VC), which was never directly identified.^[^ ^56^ ^]^ Supporting this hypothesis is the fact that none of the found polycyclic structures are observed in the base electrolyte without VC treated at the same conditions. One exemplary VC‐based structure is depicted in **Figure** **3**. It is observed in MS^1^ as a direct degradation product of the aged electrolyte and its supposed sum formula C_9_H_12_O_6_ leaves a DBE number of four and cannot be categorized into one of the literature‐known compound classes previously mentioned. Without the formation of polycyclic structures, the DBE of four would imply the formation of two additional double bonds within the molecule, which seems unlikely for the polymerization reactions taking place during battery operation. Here, the formation of (VC)_n_ species seems most likely for the observed structures as they are in line with the observed DBEs without the formation of additional double bonds within the molecule.

**Figure 4:**
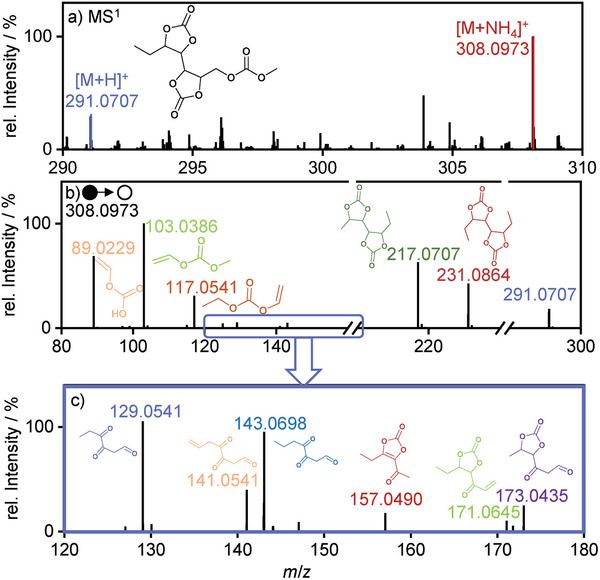
MS/MS‐experiment of the proposed (VC)_2_‐carbonate oligomer with m/z 308.0793 [M+NH_4_]^+^. In a) the m/z of the molecule are observed as [M+H]^+^ and [M+NH_4_]^+^. In b) the MS^2^‐data is shown with c) the closer examination of the m/z‐range 120–180. All postulated structures in the MS^2^‐spectra were observed as [M+H]^+^. For simplicity reasons only the uncharged structures are presented.

In **Figure** **4**, the results of the MS^2^‐experiments of a (VC)_2_‐carbonate oligomer are presented. Here, the precursor ion with *m*/*z* 308.0973 [M+NH_4_]^+^ is observed (Figure 4a). The postulated sum formula C_11_H_14_O_9_ has a relative mass deviation of <1 ppm (*m*/*z*_calc._ 308.0976 [M+NH_4_]^+^) and five DBE. A large number of fragments that indicate the presence of VC subunits are observed (Figure 4b). Here, the structure suggestion (Figure 3)is likely to be observed as the obtained fragment ion at *m*/*z* 217.0707 [M+H]^+^. There are a number of fragments further indicating the presence of VC units by decarboxylation of the precursor molecule. These are shown in the closer examination of the *m*/*z* range 120–180 (Figure 4c).

**Figure 5:**
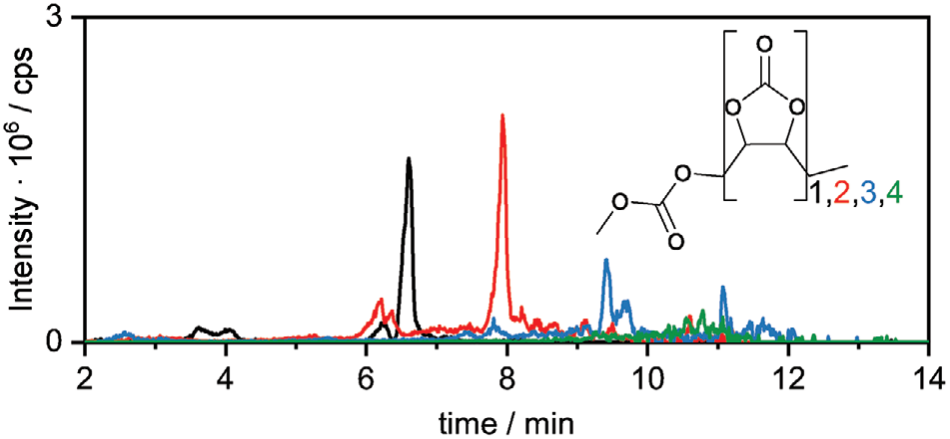
EICs of the (VC)_1‐4_‐Carbonates identified by RP‐HPLC‐MS experiments.

In addition to the identified (VC)_2_‐carbonate illustrated in Figure 4, analogous structures with one and three VC units are observed. Due to the rigid and planar ring structure of the VC units, steric effects need to be considered for polarity changes within the molecule and therefore varying retention times in RP‐HPLC. While RP‐HPLC experiments can indicate the approximate polarity of a given molecule, diastereomers of the same molecule can exhibit different polarities impossible to determine by means of HPLC. However, due to the limited number of possible reaction product classes that can be formed within the cycled LIB and the recognition of trends, that is, the addition (or subtraction) of a VC unit (*m*/u 86.0004) in the example given in **Figure** **5**, the HPLC‐MS data is considered to be sufficient. While (VC)_1_‐carbonate exhibits a relatively narrow signal in the RP chromatogram, the addition of further VC units leads to the appearance of additional signals and a broadening of the main signal, which is in good accordance with the aforementioned effect of isomeric compounds.

**Figure 6:**
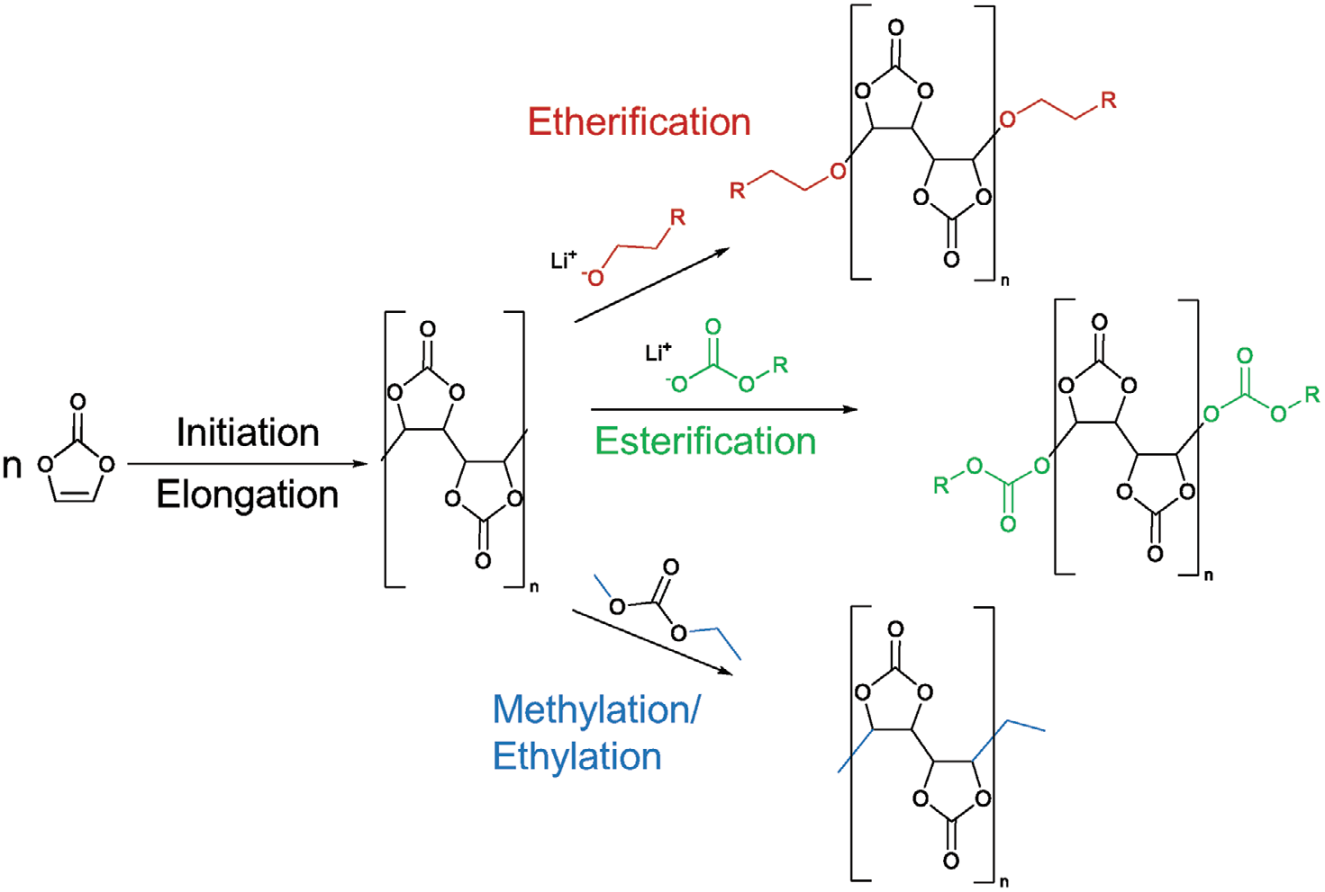
Observed VC degradation products indicating the different termination reactions (etherification (red), esterification (green) by LEMC, and methylation/ethylation (blue) by EMC) for oxidative VC polymerization identified by HPLC‐MS.

Further compounds with (VC)_n_ incorporation are identified. These include carbonate‐ether‐co‐oligomers with up to three carbonate/ether units and up to three VC units. The possibility for longer chains could not be investigated as the chromatographic resolution with each addition of carbonates and VC units. In total, 48 different VC‐based degradation products were identified with the possibility of further constitutional isomers. While the formation of polyVC has long been postulated in literature, it has, to the best of our knowledge, never been directly detected within the LIB‐electrolyte. The direct identification of polyVC‐containing molecules by means of HPLC‐MS presented in this study confirms the postulations. However, while the presented data indicates the presence of these polymerization products, it does not provide information on the mechanism. Here, only the reaction products after termination are observed. According to the presence of characteristic end groups (ether, ester, methyl/ethyl), the termination of the polymerization should be initiated by EMC and its reactive degradation products like lithium ethoxy‐monocarbonate.^[^ ^71^ ^]^ An overview of the possible termination reactions can be seen in **Figure** **6**.

With the strong indication of polyVC formed through VC oxidation, directly identified for the first time by means of HPLC‐MS, the hypothesis of the reductive formation of polyVC is also supported. As postulated by Gasteiger et al., the oxidative degradation of VC leads to polyVC similar to the reductive generation of polyVC postulated by Ota et al.^[^ ^56^, ^58^ ^]^ Therefore, approximations for the VC‐based polymerization products, in regards to the incorporation of other electrolyte components (EC, EMC, and LiPF_6_‐based degradation product) in SOTA LIBs should be possible by the investigation of the high‐voltage LIBs. The early termination of the VC polymerization could be explained by the fact that higher voltages lead to more reactions, especially of an oxidative nature, which leads to a greater possibility of termination due to parasitic reactions.

The identification of polyVC has always been difficult due to the fact that non‐soluble and long‐chained polymers cannot easily be detected by surface analysis methods (e.g., LDI‐ToF‐MS) within the SEI. Here, the surrounding matrix (i.e., the composition of the SEI, comprised of numerous inorganic and organic compounds) causes complex mass spectra with no separation of the analytes. Therefore, a characterization of not only polyVC but also further, poly‐VC‐containing compounds that are incorporated within the SEI, is extremely difficult by surface analysis of the electrodes. However, with poly‐VC being observable within the electrolyte, the excellent separation capabilities of HPLC‐MS analysis can be utilized and could give further insights into the film‐forming process by identification of further VC‐containing compounds that could be incorporated within the SEI. In our previous study, we found that the addition of VC (and fluoroethylene carbonate (FEC)) drastically lowers the toxicity of thermally aged electrolytes by suppressing of organo(fluoro)phosphate (O(F)P) formation. We postulated an incorporation of the central (F)P‐headgroup into the VC(/FEC)‐based polymer resulting in insoluble or sterically hindered compounds, thus rendering them unharmful.^[^ ^48^ ^]^ With soluble poly‐VC compounds, we can now further support this hypothesis.

### 2.2 Organo(fluoro)phosphates Incorporated into the PolyVC Polymer Film

**Figure 7:**
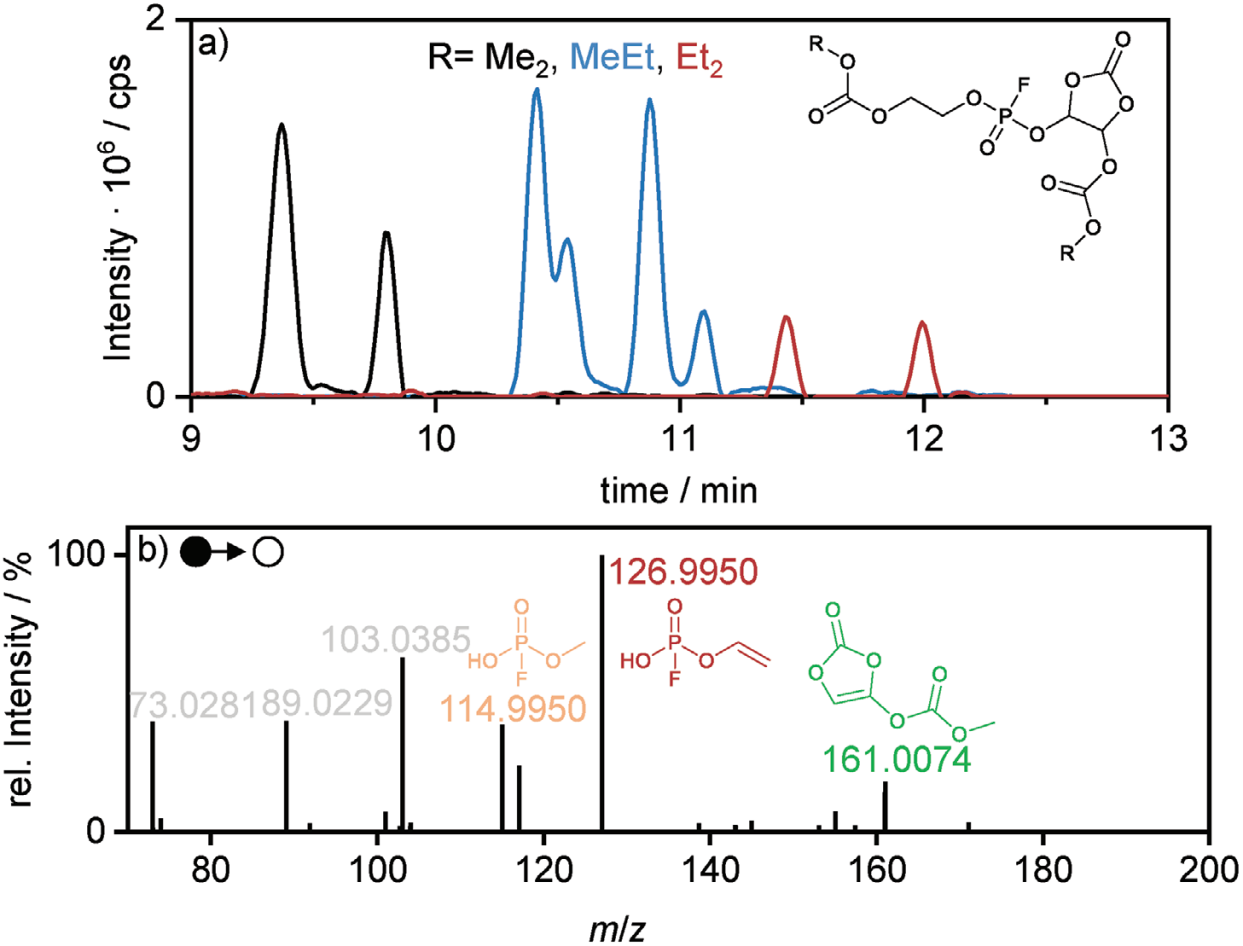
a) EICs of the identified FP‐VC carbonate co‐oligomers and b) exemplary MS^2^ fragment spectrum of MeEt‐VC‐OFP (m/z 377.0280, [M+H]^+^) with its characteristic fragments colored in orange, red, and green as identified by HPLC‐MS.

As presented in our recent study, the toxicity of different thermally aged electrolytes can be influenced by the variation of additives.^[^ ^48^ ^]^ Here, the utilization of an established method to evaluate the toxicity of fluorophosphate, which is based on the inhibition evaluation of acetylcholinesterase (AChE) indicated a lower toxicity for electrolytes which include film‐forming additives (VC, FEC) compared to the standard electrolyte. Based on these findings, a correlation of the polymerization capability of VC/FEC and the suppression of OFP‐formation is postulated. Here, we propose that the film‐forming additives should be able to incorporate the central fluorophosphate headgroups (O_3_H_2_FP) into the polymerized decomposition products being part of the SEI, rendering them less toxic due to a) steric hindrance regarding their size if in solution and b) immobilizing them within the SEI (and CEI). With the soluble VC polymerization products reported above, this hypothesis could be further supported by identifying OFP degradation products incorporated in polyVC subunits. Evaluation of the chromatographic and mass spectrometric data indeed indicates the presence of VC‐containing OFPs. **Figure** **7** displays an exemplary RP‐HPLC chromatogram a) of three different molecules with either Me_2_‐/Et_2_‐ and MeEt end groups as observed in all previously found OFPs. Furthermore, for MeEt end groups, the characteristic shoulder in the signal is observed. The double signals for each molecule could be explained by the aforementioned diastereomers. The general retention times are in good accordance with the literature‐known OFPs and further support the identification.^[^ ^72^ ^]^ In the MS^2^ spectrum b) of the MeEt‐VC‐OFP (*m*/*z* 377.0280 [M+H]^+^), the central methylated and ethylated fluorophosphate head groups are observed at *m*/*z* 114.9950 and *m*/*z* 126.9950 ([M+H]^+^), respectively. The fragment indicating the presence of a VC unit is observed at *m*/*z* 161.0074 ([M+H]^+^). The typical, carbonate‐indicating fragments are observed at *m*/*z* 73.0281, 89.0299, and 103.0385, each as [M+H]^+^ ion.

A total number of 24 different FP‐VC‐carbonate co‐oligomers were identified. These include up to two VC units with a possibility of additional units, which could not be unequivocally identified due to their increasing variation in retention times. In addition, up to three carbonate sidechains are observed for molecules with one and two VC units. The same structures are found for phosphates without fluorine. These findings strongly indicate an incorporation of FP headgroups into the VC‐based polymer films. They also support the hypothesis that film‐forming additives have a substantial impact on the toxicity of LIB electrolytes as these products could be incorporated within the SEI. While quantitative analysis could provide the exact concentration of OFPs present in the electrolyte, the toxicological assessment yields sufficient, and arguably more important information on the amount of OFPs present within the electrolyte.

**Figure 8:**
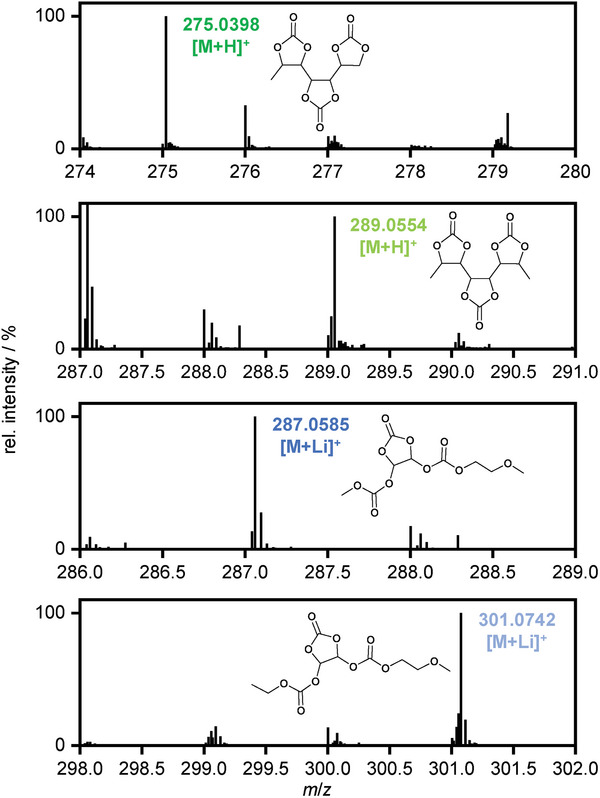
LDI mass spectra of VC degradation products observed on the CEI of the cathode after the first ten charge/discharge cycles.

This study presented the early life behavior of the LIB after the formation of the cell (10 cycles of charge/discharge) and therefore should give a good indication of the time trajectory of possible reactions during further cycling. However, long‐term studies and end‐of‐life investigations of this phenomenon need to be conducted to ensure the improved safety of LIBs regarding their toxicity over the whole life span of the battery.

### 2.3 Poly‐VC on the Cathode Electrolyte Interphase

With the structure elucidation of poly‐VC degradation products within the electrolyte by means of HPLC‐MS, a targeted analysis of the SEI/CEI by means of LDI‐MS is enabled. For the anode side (i.e., the SEI) no identification of polyVC or VC‐OFPs was possible, as the experiments do not yield any comprehensive data. Possible explanations are the aforementioned matrix effects including ion suppression. As the CEI should be less developed and differently comprised than the SEI, together with the fact that VC is the only oxidatively degraded compound in the employed electrolyte, there are less matrix effects, and therefore a conclusive analysis of the CEI is enabled.^[^ ^73^ ^]^ For cells charged to a UCV of 4.8 V, an increased amount of degradation products is found on the cathode surface. Among these degradation products, poly‐VC species similar to those found within the electrolyte are observed (**Figure** **8**). The observation of solid and polymerized poly‐VC species on the cathode side proves the oxidative film formation on the cathode side at high‐voltages caused by VC. However, no VC‐OFP co‐oligomers are observed on the CEI, indicating the absence of OFP precursors like difluorophosphate at the cathode side. This is in good accordance with the literature, as the generation of these species is postulated to be of a reductive nature and therefore expected to solely be present at the anode side of the cell.^[^ ^74^ ^]^

**Figure 9:**
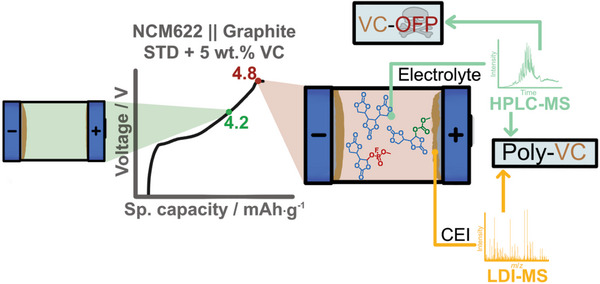
Schematic summary of the electrode crosstalk triggered by VC‐oxidation at UCV of 4.8 V. VC forms PolyVC, which is observed on the CEI and within the electrolyte. VC‐OFP co‐oligomers are solely formed within the electrolyte indicating an electrode crosstalk as OFP‐precursor molecules are reductively formed at the anode side.

OFP‐VC co‐oligomers are solely observed in the electrolyte at a UCV of 4.8 V and are non‐detectable (as well as poly‐VC) in the electrolyte in cells cycled under low UCVs (<4.3 V). Therefore, it can be assumed that electrode crosstalk is responsible for the formation of OFP‐VC co‐oligomers, where the oxidatively generated poly‐VC or the reductively generated OFP‐precursors should migrate toward the anode or cathode side, respectively, to finally form the VC‐OFP degradation products. However, further research has to be conducted to confirm the exact mechanism(s) responsible for the formation of OFP‐VC co‐oligomers. Here, the reductive formation of polyVC is of the highest interest, as this reaction would take place on the anode side, where it could then directly incorporate the reductively generated OFP precursors. However, method development for LDI‐MS analysis has to be conducted to overcome challenges like the high complexity of the SEI‐composition and the consequential ion suppression.

## 3 Conclusion

In this study, the oxidative decomposition of vinylene carbonate (VC)‐based electrolytes are investigated in LIB cells at a high upper charge voltage (UCV) of 4.8 V. Through the termination of the oxidative polymerization in the early stages (with reactive species generated at high‐voltages), it is possible to analyze the still soluble degradation products via extraction of the electrolyte from the LIB followed by high‐performance liquid chromatography‐mass spectrometry (HPLC‐MS) analysis, while the cathode electrolyte interphase (CEI) can be analyzed by LDI‐ToF‐MS (**Figure** **9**).

Through oxidization, reductive polymerization (and subsequent precipitation) of VC is mitigated to investigate possible SEI‐forming compounds and therefore gather a deeper understanding of the film‐forming processes (CEI and SEI). Here, the long‐postulated, but not so far directly detected poly‐VC is observed in operando for the first time, proving the oxidative polymerization of VC resulting in this species. Moreover, different routes of termination reactions are presented. A total of 48 polyVC degradation products with varying end groups (carbonate, ethoxy, and methyl/ethyl) are identified. With the identification of VC polymerization products that form the SEI/CEI, the postulation from our previous work, that toxic OFPs are bound by film‐forming additives and are incorporated within the SEI, rendering them unharmful, is evaluated. Indeed, OFP‐VC molecules are observable. A total of 24 VC‐OFP degradation products have been identified, thus supporting the hypothesis of SEI‐bound OFPs. The oxidative formation of VC‐OFPs indicates an electrode crosstalk as OFP precursor molecules, for example, difluorophosphate and monofluorophosphate, are reductively generated at the anode side. Therefore, VC‐OFP co‐oligomers can only be generated via migration of either polyVC degradation products or OFP precursors toward the anode or cathode, respectively. With evidence of VC‐OFPs being observable reaction products, the previous finding is supported that the general toxicity of the electrolyte is lowered by OFP‐incorporation into the VC‐polymer film (CEI and SEI) is supported. With that, the need for film‐forming additives is further reinforced as they not only lead to better battery performance, but also lower the general toxicity of the LIB‐electrolyte.

## 4 Experimental Section

Identification of soluble electrochemically induced electrolyte decomposition products was performed on an UHPLC system UltiMate 3000 (Thermo Scientific, Dreieich, Germany) equipped with a WPS‐3000 autosampler module, a TCC‐3000SD column oven, an SRD‐3600 degasser, and a WPS‐3000 dual gradient pump module. Reversed‐phase chromatography was conducted on a Poroshell 120 SB‐C18 column (dimensions,100 × 2.1 mm, particle size, 1.9 µm; manufacturer, Agilent Technologies, Santa Clara, CA, USA) at 40 °C and a flow rate of 0.5 mL min^−1^ The mobile phase gradient consisted of water with 0.1 % formic acid (A) and acetonitrile (B). The employed gradient started with 5% B from 0 to 1.9 min and increased to 60 % within 12.1 min, where it was kept constant for 6 min. Finally, the column was equilibrated at 5 % B for 4 min. The Q Exactive Plus Hybrid Quadrupole‐Orbitrap mass spectrometer (Thermo Fisher Scientific, Waltham, MA, USA) in combination with a HESI‐II probe was utilized in positive ionization mode for the detection. HESI settings were chosen as follows: heater temperature 300 °C, sheath gas flow rate 45 auxiliary units (AU), auxiliary gas flow rate 15 AU, sweep gas flow rate 1 AU, spray voltage + 3 kV. The mass spectrometer was operated in full scan mode with a resolution of 140 000 (FWHM, at *m*/*z* 200). Other settings were: *m*/*z* range 150–900, s‐lens RF level 80, AGC target 1e6, maximum injection time 100 ms. Data‐dependent MS^2^ experiments were carried out in an HCD cell with a normalized collision energy of 30 eV (based on an *m*/*z* of 500) and a resolution of 17 500 (at *m*/*z* 200). The AGC target was set to 1e5, the maximum injection time to 50 ms, and the quadrupole isolation window ± 1.0 Da. The system was operated with Xcalibur 4.6 software with the SII Chromeleon plugin.

A timsToF flex (Bruker Daltonics, Bremen, Germany) mass spectrometer equipped with a 10 kHz frequency tripled Nd:YAG laser (355 nm) was used for LDIToF‐MS and LDIToF‐tandem MS (MS/MS) analyzes of the SEI/CEI. Single LDIToF‐MS and LDIToF‐MS/MS spectra were recorded by summation of 12 spots distributed over the surface of the analyzed electrode. The mass spectrometer was controlled by timsControl 3.0.20.0 (Bruker Daltonics, Bremen, Germany). LDIToF‐MS and LDIToF‐MS/MS data were evaluated using DataAnalysis 6.0 software (Bruker Daltonics, Bremen, Germany).

### Chemistry and Materials

The electrolytes employed in this study contained EC and EMC (30/70 wt.%) with 1 mol L^−1^ LiPF_6_ as conducting salt (standard electrolyte (STD)) as well as 5 wt.% VC as film‐forming additive. The electrolytes were acquired from Solvionic (Toulouse, France). Acetonitrile (ACN, LC‐MS grade, VWR International, Darmstadt, Germany) and deionized water (18.2 MΩ cm^−1^, Millipore, Molsheim, France) were used for sample dilution and as eluents for LC‐MS based analysis. All chemicals were used as received.

### Cell Assembly

Two electrode coin cells^[^ ^75^ ^]^ (CR2032) were assembled and used for the investigation of the electrolyte after ten cycles of battery operation. Triple layered polypropylene separators (FS2190, Freudenberg, Weinheim, Germany) were soaked with 150 µL of the aforementioned electrolytes. The coin cells were assembled in a dry room (dew point of −65 °C, H_2_O <10 mL m^−3^). After electrochemical operation, the coin cells were opened in a dry room and the electrodes were taken out and washed with 100 µL of EMC (Solvionic, Toulouse, France) before attaching them to indium tin oxide (ITO)‐coated microscopic glass slides (70–100 Ω sq^−1^, Sigma–Aldrich, Steinheim, Germany) using conductive double‐sided adhesive tape. The sample slides were mounted to the sample carrier in an Ar‐flushed airlock and inserted in the evacuated (≈2.6 mbar) LDI source of the mass spectrometer for analysis without contact to ambient air. Electrolyte extraction was conducted via centrifugation of the separators. The samples were diluted 1:100 v/v with ACN prior to analysis.

### Electrochemical Operation

A variety of different charge/discharge programs were applied to the assembled coin cells through connection to a MACCOR system (CellCare Technologies, Market Harborough, United Kingdom). All experiments consisted of three charge/discharge cycles at 0.1 C (formation period) and seven cycles at 1  C (1  C corresponds to 178 mA g^−1^). Here, the cut‐off voltages varied from 3.2 to 4.2, 4.5, and 4.8 V with constant current constant voltage (CCCV) charge and constant current (CC) discharge cycles with cut‐off voltage of 2.8 V.

## Conflict of Interest

The authors declare no conflict of interest.

## Data Availability

Research data are not shared.
